# The Administration of Anæsthetics

**Published:** 1919-12-13

**Authors:** 


### Conclusions.

### THE ADMINISTRATION OF ANÆSTHETICS.

In the City Coroner's Court, Dr. F. J. Waldo recently opened an inquest on the body of Robert Warner, aged sixty-nine, a baker and confectioner, lately carrying on business in New North Road, Islington, who died in St. Bartholomew's Hospital during an operation by Sir D'Arcy Power for hernia. Commenting on the fact that at some hospitals students were allowed to administer anæsthetics, the coroner said St. Bartholomew's Hospital had three resident anæsthetists and four honorary visiting ones, and they never allowed students or nurses to give anæsthetics. Many years ago Dr. Waldo suggested legislation on the subject, but although Lord Gladstone, then Home Secretary, ordered a departmental committee to investigate the subject, and very important recommendations were made in 1910, nothing had been done to give effect to them. The coroner also told the jury that at a recent inquest held by him on a child whose death was accelerated by chloroform given by a medical student for an operation in a general hospital, the same student had also been giving anæsthetics for operations at a special hospital in South London, and a general one in North London, owing, as alleged, to the scarcity of properly qualified medical men.

Quoting from the late Sir Frederic Hewitt's work on "Anæsthetics and their Administration" (1907), the coroner read the following: "Legislation is required restricting the administration of anæsthetics, save in the most exceptional circumstances, to duly registered medical practitioners, and separating the responsibilities of the anæsthetist from those of the operator," and "curiously enough, there are still many thousands of educated men and women who look upon the process of anæsthetising, as practised by the now large body of special anæsthetists, as corresponding in its importance and responsibilities to poulticing, bandaging, or some other purely mechanical procedure, and who have little or no idea that the success of even the simplest surgical operation may be dependent to a large extent upon the anæsthetist."

The coroner added that he had written an article on "Deaths under Anæsthetics," published in the *Medical Press and Circular*, March 18, 1908, and in the *Lancet*, October, 1908, in which the first (of six) conclusions drawn by him was to the effect that present available data as to deaths during anæsthesia are so imperfect as to be useless for the purposes of formal investigation.

Among his eight recommendations were the following: (1) That no general or local anæsthetic shall be administered by any but a duly qualified medical man, except under most exceptional circumstances, which shall be duly reported to some recognised official authority. (2) That so far as possible special skilled anæsthetists be appointed to all hospitals and infirmaries, and that resident anæsthetists be provided in the larger institutions. (3) That notification be made to the coroner of all deaths occurring at any stage of general anæsthesia by the anæsthetist or by others concerned. (4) That a Royal Commission with advisory powers might with advantage be appointed to inquire into the present facts of deaths under anæsthesia, together with the official machinery available for registration.

The Departmental Committee referred to was appointed in December, 1908, and made various valuable recommendations in a report dated March 18, 1910, among which were the following:

(1) No general respirable anæsthetic should be administered by any person who is not a registered medical or dental practitioner.

(2) Registered dentists should be confined to the use of nitrous oxide gas (laughing gas) for dental operations, and should not employ the general respirable anæsthetics of longer duration.

(3) Intra-spinal anæsthesia should be practised only by registered medical practitioners.

(4) In the case of any death under an anæsthetic in a hospital or other similar institution, there should be a scientific investigation into the actual cause of death conducted by the authorities of the institution.

(5) A small standing scientific committee on anæsthetics should be instituted under the authority of the Home Office.

So far as he (the coroner) knew, none of these recommendations had yet been carried out, and, as the law at present stands, anæsthetics may still be administered by wholly unqualified persons—by anybody, in fact.

